# *Glycyrrhiza uralensis* Polysaccharide Gold Nanoparticles as Antigen Carriers and Potential Adjuvant to DC Vaccines

**DOI:** 10.3390/pharmaceutics17091213

**Published:** 2025-09-17

**Authors:** Yanan Zhao, Ming Song, Yilizilan Dilixiati, Shanshan Cai, Reyilanmu Maisaidi, Patanmu Aili, Jinyao Li, Lili Han, Adila Aipire

**Affiliations:** 1 Xinjiang Key Laboratory of Biological Resources and Genetic Engineering, College of Life Science and Technology, Xinjiang University, Urumqi 830017, China; zhaoyanan514@163.com (Y.Z.); 107552100985@stu.xju.edu.cn (M.S.); 107552100963@stu.xju.edu.cn (Y.D.); 18699927521@163.com (S.C.); 17690025193@163.com (P.A.); ljyxju@xju.edu.cn (J.L.); 2 Department of Gynecology, People’s Hospital of Xinjiang Uygur Autonomous Region, Urumqi 830001, China; 18814115890@163.com

**Keywords:** GUPS-AuNPs, DC vaccine, immune enhancement, antigen delivery, antitumor

## Abstract

**Background**: Cervical cancer is the fourth leading cause of death among women worldwide, with human papillomavirus (HPV) identified as a major contributing factor. This study investigates the immunostimulatory activity and antigen delivery efficiency of *Glycyrrhiza uralensis* polysaccharide gold nanoparticles (GUPS-AuNPs) and assesses the antitumor efficacy of an HPV dendritic cell (DC) vaccine using GUPS-AuNPs as a delivery system. **Methods**: GUPS-AuNPs were synthesized via a green reduction method and characterized using advanced techniques, including SEM, EDS, TEM, UV, and FT-IR spectroscopy. DCs served as the primary experimental model, with flow cytometry employed to evaluate the immunostimulatory activity and antigen delivery effectiveness of GUPS-AuNPs. Additionally, a TC-1 tumor-bearing mouse model was established to assess the immunostimulatory and antitumor effects of the HPV-DC vaccine facilitated by GUPS-AuNPs. **Results**: The synthesized GUPS-AuNPs exhibited a particle size of 120.77 ± 3.13 nm, a surface charge of −11.9 ± 2.1 mV, and excellent stability. Flow cytometry analysis demonstrated that GUPS-AuNPs significantly enhanced DC maturation and promoted T cell proliferation. Furthermore, antigen delivery experiments revealed that GUPS-AuNPs improved the antigen capture capabilities of DCs. Confocal imaging confirmed that GUPS-AuNPs extended the intracellular retention time of antigens. In vivo studies showed that the HPV-DC vaccine formulated with GUPS-AuNPs as carriers effectively suppressed tumor growth, elevated the populations of CD4^+^ T and CD8^+^ T cells in the spleen, and induced a robust antigen-specific immune response. **Conclusions**: GUPS-AuNPs effectively enhance DC maturation and antigen delivery, significantly boosting the adaptive immune response triggered by HPV vaccines and leading to the inhibition of tumor progression. This research introduces GUPS-AuNPs as a novel, safe, and efficient antigen delivery platform with promising potential for vaccine development.

## 1. Introduction

Cervical cancer is the fourth most common malignant tumor among women and a major cause of cancer-related mortality, ranking as the second leading cause of cancer deaths in females. Consequently, it poses a serious threat to women’s health [1,2]. Persistent infection with HPV is recognized as the primary cause of cervical cancer, with high-risk strains, especially HPV 16 and 18, responsible for over 70% of cases [3]. While surgical intervention remains the mainstay of cervical cancer treatment, the development of therapeutic vaccines targeting HPV presents a valuable complement to surgical approaches, improving treatment outcomes and reducing the likelihood of recurrence. This promising strategy offers a significant opportunity to prevent both the onset and progression of cervical cancer, providing renewed hope to patients affected by the disease [4].

DCs are specialized antigen-presenting cells that function as professional carriers for antigen delivery. DC vaccines work by capturing antigens ex vivo and then reintroducing them into the body to activate and guide the antitumor responses of tumor-killing T cells [5], showcasing significant therapeutic potential in cancer treatment. However, these vaccines face several challenges, including limited antigen delivery efficiency and inadequate immunogenicity. Consequently, enhancing the immune responses of DC vaccines requires the optimization of antigen delivery mechanisms and the incorporation of adjuvants to amplify immunogenicity [6]. Research has shown that certain naturally derived polysaccharides, when used as adjuvants to induce mature DC tumor vaccines, possess strong targeting capabilities and high stability, leading to more effective tumor cell elimination [7,8].

Previous research by our group has demonstrated that GUPS enhance macrophage activation through the TLR4 receptor pathway, promote DC maturation, and serve as an effective vaccine adjuvant, boosting both antibody and cellular immune responses across a range of vaccine types, including protein vaccines, DNA vaccines, and HPV-DC vaccines [9]. However, natural polysaccharides face inherent challenges, such as low bioavailability, short half-life, and limited stability, which constrain their clinical applications [10]. Gold nanoparticles (AuNPs), defined as particles ranging from 1 to 100 nm in size, offer several advantages, including straightforward synthesis, strong binding capabilities, and excellent biocompatibility, making them powerful vehicles for drug delivery [11]. Combining polysaccharides with gold nanoparticles has been shown to enhance the immunostimulatory properties of polysaccharides, resulting in improved tumor therapeutic effects, while also capitalizing on the targeting, photothermal, and carrier attributes of AuNPs [12]. Building on this synergy, the present study leveraged GUPS as a reducing agent to green-synthesize GUPS-AuNPs. We characterized their physicochemical properties and evaluated their immunoactivity and adjuvant effects. Notably, our findings revealed that GUPS-AuNPs demonstrated significantly higher immunostimulatory activity, prolonged intracellular antigen retention, and enhanced antitumor efficacy when compared to GUPS alone, particularly in the context of the HPV-DC vaccine.

## 2. Materials and Methods

### 2.1. Materials and Reagents

Ural licorice, sourced from in the Xinjiang Uygur Autonomous Region, Yining, China, was processed to extract Glycyrrhiza uralensis polysaccharides (GUPS), which were subsequently preserved in the laboratory, as verified by Xinjiang Pharmaceutical Research. Additionally, the HPV-containing plasmid pGEX-2T-HPV 16 E7-DH5α and TC-1 cells were stored at the Xinjiang Key Laboratory of Genetic Engineering for Bioresources, affiliated with Xinjiang University.

HAuCl_4_ and fluorescence-labeled chicken ovalbumin were procured from Yuanye Biologicals (Shanghai, China). The flow-through antibody was supplied by Elabscience (Wuhan, China), while recombinant granulocyte-macrophage colony-stimulating factor was obtained from Peprotech (Rocky Hill, NJ, USA). The red fluorescence cell membrane staining kit (DiI), lysosomal red fluorescence probe (Lyso-Tracker Red), and anti-fluorescence quenching sealing solution (containing DAPI) were sourced from BiyunTian (Shanghai, China). Lipopolysaccharide (LPS) was purchased from Sigma-Aldrich (St. Louis, MO, USA). The protein transport inhibitor, Golgistop™, as well as the cell membrane-disrupting fixative and detergent, were acquired from BD Biosciences (San Jose, CA, USA). Furthermore, the CellTrace™ Violet cell proliferation kit was supplied by Thermo Fisher (Waltham, MA, USA).

### 2.2. Synthesis of GUPS-AuNPs

Begin by placing a 250 mL round-bottom flask on a magnetic stirrer set to a consistent stirring speed of 400 rpm. Add 10 mL of a 0.01% HAuCl_4_ solution to the flask. Heat the flask in a boiling water bath for 5 min, then introduce 50 mL of a 1 mg/mL GUPS solution, ensuring the GUPS-to-0.01% HAuCl_4_ ratio is maintained at 5:1. Continue stirring the mixture at boiling temperature for 15 min, or until the solution’s color stabilizes. Once this step is complete, rapidly cool the solution to room temperature by centrifuging it in an ice bath at 18,000 rpm for 10 min. After cooling, perform a second centrifugation under the same conditions (18,000 rpm for 10 min), carefully aspirating and discarding the supernatant. Rinse the resulting precipitate with ultrapure water and allow it to air dry. The final product obtained is designated as GUPS-AuNP.

### 2.3. Physicochemical Characterization of GUPS-AuNP

The transmission electron microscopy (TEM) analysis was performed on GUPS-AuNP dissolved at a concentration of 1 mg/mL. The morphology of the material was characterized using TEM, while the elemental distributions were effectively visualized with mapping technology, offering a clear and intuitive representation.

Field emission scanning electron microscopy (SEM) and energy-dispersive X-ray spectroscopy (EDS) were utilized to examine the morphological features and elemental composition of GUPS-AuNP dry powder. These analyses were performed using a scanning electron microscope (model SU8010). Additionally, UV–visible absorption spectroscopy was utilized to analyze GUPS, AuNPs, and the GUPS-AuNP composite, with the wavelength scanning range set between 190 and 900 nm, which includes both the ultraviolet and visible light regions.

Infrared spectroscopy (FT-IR) analysis was performed by homogenizing 2 mg of GUPS-AuNP with 400 mg of KBr to create a sample pellet. The pellet was scanned using an FT-IR spectrometer at a resolution of 4 cm^−1^, covering a wavenumber range of 4000 cm^−1^ to 400 cm^−1^. The analysis was conducted using OPUS 6.5 software, with a total of 64 scans recorded.

ζ-Potential analysis: The particle size of GUPS-AuNP was measured using a laser particle size analyzer, with three parallel tests conducted to ensure accuracy.

### 2.4. Bone Marrow-Derived DC Induction Culture

Granulocyte-macrophage colony-stimulating factor (GM-CSF) was employed to induce dendritic cells (DCs) from the bone marrow of C57BL/6 mice. The procedure involved the extraction of bone marrow from the femurs and tibias of the mice, followed by centrifugation at 1200 rpm for 7 min to remove the supernatant. The resulting cells were resuspended and cultured in RPMI-1640 complete medium, which was supplemented with 10% fetal bovine serum, 1% antibiotics, and 20 ng/mL GM-CSF. Cultures were maintained in 60 mm Petri dishes at 37 °C in a 5% CO_2_ environment. On the second and fifth days of culture, half of the medium in each dish was carefully removed and replaced with fresh medium. Similarly, on the third day, unattached cells were aspirated, and the medium was refreshed to ensure continued cell growth.

### 2.5. Detection of DC Surface Molecules by Flow Cytometry (FCM)

On the seventh day of culture, DCs were harvested and seeded into 24-well plates at a density of 2 × 106 cells per well. The effects of GUPS-AuNP treatment at varying concentrations on DCs were evaluated over a 12 h period, with 20 ng/mL LPS serving as a positive control. After treatment, the cells were collected by centrifugation at 1200 rpm for 7 min, and the supernatant was preserved for cytokine concentration analysis. The cells were then rinsed with PBS, stained with fluorescent antibodies targeting CD40 and CD86 for 15 min, and resuspended following a subsequent PBS wash. Finally, the cell suspension was filtered through a 200-mesh copper mesh into a collection tube for flow cytometry assessment.

### 2.6. Enzyme-Linked Immunosorbent Assay (ELISA) for Assessing Cytokine Secretion Levels

The DC culture supernatant was analyzed for cytokine content using an ELISA kit, following the manufacturer’s instructions (Elabscience). The procedure was conducted as follows: Cytokine standards (2000 pg) were prepared using a sample diluent and subjected to gradient dilution to create seven standard concentrations: 1000 pg/mL, 500 pg/mL, 250 pg/mL, 125 pg/mL, 62.5 pg/mL, 31.25 pg/mL, and 15.625 pg/mL. A total of 100 μL of each appropriately diluted standard and sample solution was added to pre-coated 96-well plates and incubated at 37 °C for 90 min. After incubation, the liquid in the wells was discarded, and 100 μL of diluted antibody solution was added to each well, followed by an additional incubation at 37 °C for 60 min. The wells were then washed by adding 200 μL of washing solution to each well for 1 min, after which the liquid was removed by tapping the plate dry. This washing process was repeated three times. Afterward, 100 μL of pre-diluted (1:100) enzyme solution was added and incubated at 37 °C for 30 min. The samples underwent another incubation at 37 °C for an additional 30 min. Once this step was complete, 200 μL of washing solution was added to each well for 1 min, and the washing process was repeated five times. Finally, 90 μL of color development solution was added to each well and allowed to react for 5 to 15 min at room temperature. To stop the reaction, 50 μL of termination solution was added to each well. The optical density (OD450) value was then measured using a multifunctional enzyme marker.

The cytokine standard curves were generated based on the requirements of the experiment, with the horizontal axis (X-axis) representing the concentration and the vertical axis (Y-axis) indicating OD450:

IL-6: y = 0.0006 x + 0.0612, R^2^ = 0.9993

TNF-α: y = 0.0008 x + 0.0787, R^2^ = 0.9995

IL-12p40: y = 0.0008 x + 0.1008, R^2^ = 0.9923

### 2.7. GUPS-AuNP Antigen Phagocytosis Assay

On day 7, DCs were exposed to GUPS-AuNP at a concentration of 2 μg/mL or LPS at 20 ng/mL for 12 h. Afterward, the cultures were incubated for 1 h at 37 °C in a 5% CO_2_ environment. Phagocytosis was then interrupted by adding 50 μg/mL of FITC-Dextran to the culture. To fully terminate phagocytosis, pre-chilled PBS was subsequently introduced. The cells were collected, washed twice with PBS, and the cell pellet was obtained through centrifugation.

### 2.8. Evaluation of the Impact of GUPS-AuNP-Treated Dendritic Cells on T Cell Proliferation

DCs from primary cultures were harvested on day 7 and exposed to 2 μg/mL of GUPS-AuNP and GUPS for 12 h, respectively. LPS at a concentration of 20 ng/mL was used as the positive control. Spleens from BALB/c mice were homogenized, followed by the addition of red blood cell lysate. Spleen cells were then isolated through centrifugation and subsequent washing with PBS. These cells were stained with 1 μM CellTrace™ Violet, rinsed with PBS, and resuspended in complete medium. Spleen cells were paired with DCs at a ratio of 5:1, with the spleen cells set at a density of 1 × 10^6^ cells per well. The two cell populations were combined and incubated in a final volume of 1 mL. After 72 h, the cells were collected, and the proliferation of CD4^+^ T cells and CD8^+^ T cells was assessed using FCM.

### 2.9. Binding of GUPS-AuNP to OVA Proteins

In a 50 mL round-bottom flask, 10 mL of GUPS-AuNP was mixed with 100 μL of FITC-OVA. The mixture was stirred in the dark using a magnetic stirrer for 6, 12, and 24 h. Subsequently, PBS was used to remove unbound proteins, yielding the GUPS-AuNP/FITC-OVA complex. This complex was then analyzed using a UV spectrophotometer. The binding efficiency of GUPS-AuNP to the OVA protein was evaluated using a BCA protein quantification kit, with bovine serum albumin (BSA) serving as the standard for the assay.

### 2.10. Ability of FCM to Detect DC Capture of FITC-OVA Antigen

DCs derived from primary cultures were harvested on day 7 through centrifugation at 1200 rpm for 7 min. The cells were then seeded into 24-well plates at a concentration of 1 × 10^6^ cells/mL and exposed to GUPS-AuNP/FITC-OVA under various time intervals: 12 h, 6 h, 4 h, 1 h, and 15 min. During the treatment, GUPS-AuNP/FITC-OVA was shielded from light, with free FITC-OVA serving as the control. After the treatment period, the cell pellets were collected via centrifugation, resuspended in PBS, and filtered through a 200-mesh copper screen into flow cytometry tubes for analysis. FCM was then used to determine the proportion of FITC^+^ cells.

### 2.11. Confocal Microscopy Analysis of GUPS-AuNP-Mediated Delivery of FITC-OVA

DCs were treated with GUPS-AuNP/FITC-OVA under dark conditions for various durations of 12 h, 6 h, 4 h, and 1 h while an untreated group served as the control. Following treatment, the cells were washed twice with PBS. A staining solution was prepared according to the protocol outlined in the cell membrane red fluorescent staining kit. Subsequently, 500 μL of the staining solution was added to each sample, and the samples were incubated at 37 °C for 20 min. After incubation, the samples were washed twice with PBS, and 200 μL of anti-fluorescence quenching sealing solution containing DAPI was carefully added. The samples were then incubated at 37 °C for 10 min before imaging with a Nikon A1 confocal laser scanning microscope.

### 2.12. Localization of GUPS-AuNP/FITC-OVA in DCs

DCs from primary culture were collected on day seven through centrifugation at 1200 rpm for 7 min, counted, and subsequently transferred to a specialized Petri dish for laser confocal imaging at a density of 1 × 10^5^ cells/mL in 200 µL. After a 10 min incubation, 800 µL of complete medium was added, bringing the total volume to 1 mL. The cells were then treated with GUPS-AuNP/FITC-OVA for varying durations while being protected from light, followed by washing with PBS. The working solution for Lyso-Tracker Red, a lysosomal red fluorescent probe, was prepared in accordance with the manufacturer’s instructions: a small amount of Lyso-Tracker Red was diluted in PBS at a ratio of 1:20,000, yielding a final concentration of 50 nM. One milliliter of the prepared solution was added to each sample and left to stand at room temperature for 20 min. The samples were then washed twice with PBS, after which 200 µL of an anti-fluorescence quenching sealing solution containing DAPI was introduced. Following a 10 min incubation at room temperature, images were acquired using a confocal laser scanning microscope.

### 2.13. Establishment and Treatment of TC-1 Tumor Model Mice

To assess the antitumor efficacy of the HPV DC vaccine, a TC-1 tumor model was established in mice. The procedure for creating the TC-1 tumor model and the subsequent treatment strategy is depicted in Figure 5a. During the logarithmic growth phase, TC-1 cells were collected, centrifuged at 1000 rpm for 5 min, and resuspended in PBS at a concentration of 1 × 10^6^ cells/mL. Female C57BL/6 mice, aged six to eight weeks, were subcutaneously inoculated with TC-1 cells (1 × 10^5^ cells per mouse) on the right side of their backs on day 0.

Tumor-bearing mice were randomly divided into four groups, each comprising five mice. The first group, which received untreated DC peptides, acted as the control. The second group was administered the GUPS-AuNP-DC + E7pep peptide. The third group received the GUPS-AuNP-E7pro peptide-DC peptide, while the fourth group was treated with the GUPS-AuNP-E7pro-DC vaccine.

The intradermal administration of the DC vaccine was conducted on days 5 and 12 after tumor inoculation, with a total of two immunizations delivered at 7-day intervals. The mice’s mental state was closely observed, and detailed records of tumor size changes were meticulously maintained. The length (a) and width (b) of the tumor tissue were measured every two days using vernier calipers. Tumor volume was calculated, and a tumor growth curve was plotted based on the following formula: Tumor volume (V, in mm^3^) = (a × b^2^)/2, where “a” denotes the tumor’s length and “b” its width. On the 10th day following the second vaccination, mice were euthanized in strict accordance with the guidelines prescribed by the animal ethics committee. Subsequently, tumor tissues were extracted, weighed, and photographed for further analysis.

### 2.14. FCM Analysis of Spleen Immune Cells

After grinding and processing the spleen tissue, the cells were resuspended in 5 mL of complete medium and counted. Each mouse yielded two million spleen cells, which were subsequently washed with PBS. The cells were then incubated at room temperature for 15 min with antibodies against CD3, CD19, CD49b, CD4, CD8, and CD44, all while shielded from light. Following incubation, the cells were rinsed with PBS, centrifuged at 1200 rpm for 7 min, and resuspended in PBS. They were then filtered through a 200-mesh copper mesh into flow cytometry tubes. Finally, the samples were collected using flow cytometry.

### 2.15. Tregs Cell Ratio Detection

A total of 2 × 10^6^ mouse spleen cells were collected, washed with PBS, and incubated with anti-CD4 and anti-CD25 antibodies for 15 min in the dark. Subsequently, the cells were washed again with PBS and processed following the protocol specified in the Foxp3 Fixation/Permeabilization kit. Fixative solution was then added, and the cells were incubated at 4 °C in the dark for 30 min. Following incubation, the cells were washed twice with 1 mL of PBS, resuspended in PBS, and filtered through a 200-mesh copper mesh into a tube. Finally, the prepared cell samples were analyzed using flow cytometry.

### 2.16. Detection of Antigen-Specific T Cell Immune Responses

The wells of a 24-well cell culture plate were seeded with spleen cells at a concentration of 2 × 10^6^ cells/mL in a total culture volume of 1 mL. One well was treated with 0.5 μg/mL of HPV 16 E7 peptide, while the control well remained untreated. After a 2 h incubation, 0.66 μL of GolgiStop™, a protein transport inhibitor, was added to the wells. The cells were then collected 10 h later, and the antigen-specific immune response was evaluated.

The cells were collected and incubated with anti-CD4 and anti-CD8 antibodies for 15 min, followed by a PBS wash. A membrane-permeabilizing fixative was then added, and the cells were incubated for an additional 30 min at 4 °C. Afterward, the cells were washed with 1 mL of washing buffer, and the resulting cell pellets were incubated with an anti-IFN-γ antibody for 15 min. Following this step, the cells were washed again with PBS. Finally, the cells were resuspended in PBS, filtered through a 200-mesh copper mesh, and transferred to flow cytometry tubes. The samples were subsequently collected and analyzed via flow cytometry.

### 2.17. Statistical Analysis of Data

Data analysis was conducted using GraphPad Prism 10.2. (San Diego, CA, USA) Statistical evaluations employed one-way analysis of variance (ANOVA) and the Mann–Whitney non-parametric *t*-test. A *p*-value of less than 0.05 was deemed statistically significant.

## 3. Results

### 3.1. Synthesis and Characterization of GUPS-AuNP

The synthesis of GUPS-AuNPs was carried out using varying ratios (1:1, 2:1, 5:1, 7:1, 10:1, and 15:1) of GUPS (1 mg/mL) and HAuCl_4_, designated as GUPS-AuNP-I, GUPS-AuNP-II, GUPS-AuNP-III, GUPS-AuNP-IV, GUPS-AuNP-V, and GUPS-AuNP-VI. The color of the GUPS-AuNPs gradually shifted from pink to purplish-red (Figure S1b), and absorption peak analysis revealed that the peaks for GUPS-AuNP-II, GUPS-AuNP-III, GUPS-AuNP-IV, and GUPS-AuNP-V were centered around 535 nm. This finding indicates a more consistent particle size distribution for these samples. Subsequent characterization of the particles was performed. The transmission electron microscopy results showed that the particle sizes of these four GUPS-AuNP samples ranged between 9.45 ± 1.67 nm and 10.69 ± 1.41 nm. Among them, GUPS-AuNP-II and GUPS-AuNP-III displayed a more uniform size distribution (Figure S1c). A comparison of the in vitro immunostimulatory activities of the four GUPS-AuNP samples demonstrated that both GUPS and the GUPS-AuNPs significantly upregulated the expression of CD40 and CD80. Notably, GUPS-AuNP-III exhibited a stronger effect than GUPS alone (Figure S1d,e). Future experiments will focus on evaluating the immunostimulatory activity and adjuvant effects of GUPS-AuNP-III.

**Figure 1:**
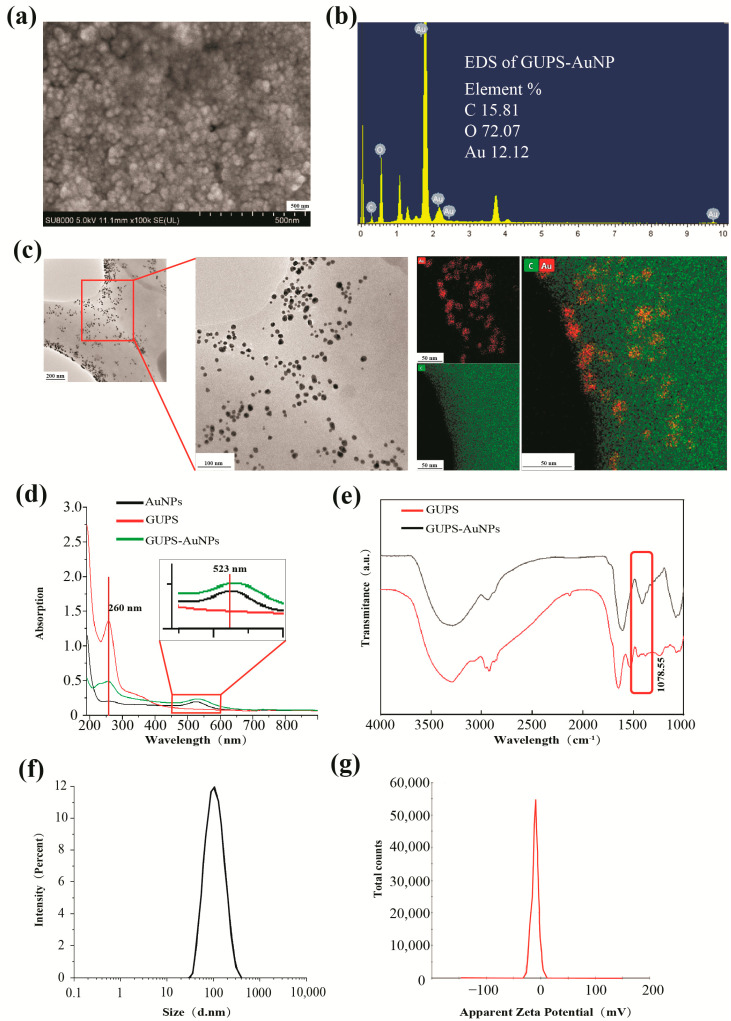
Physicochemical characterization of GUPS-AuNP: (**a**) SEM image of GUPS-AuNP; (**b**) EDS analysis of GUPS-AuNP; (**c**) TEM and mapping of GUPS-AuNP; (**d**) UV analysis results, the characteristic absorption peak of GUPS is at 260 nm, the characteristic absorption peak of AuNPs is at 523 nm, and GUPS-AuNP with both characteristic absorption peaks; (**e**) FT-IR spectra of GUPS and GUPS-AuNP; (**f**,**g**): particle size (**f**) and ζ potential (**g**) of GUPS-AuNP.

SEM was employed to characterize the structure of GUPS-AuNPs, demonstrating that they exhibit a relatively uniform structural morphology (Figure 1a). EDS was used to analyze the elemental composition of GUPS-AuNP, revealing that 15.81% of the atoms were carbon, 72.03% were oxygen, and 12.12% were gold (Figure 1b). TEM mapping further confirmed that GUPS-AuNPs exhibited a regular spherical distribution, with gold nanoparticles uniformly adsorbed onto the surface of GUPS (Figure 1c). Ultraviolet–visible (UV–Vis) spectroscopy displayed characteristic absorption peaks at 257 nm for GUPS and at 538 nm for AuNPs. The synthesized GUPS-AuNP exhibited absorption peaks at both 257 nm and 538 nm (Figure 1d), indicating the successful binding of GUPS and AuNPs. Fourier transform infrared (FTIR) spectroscopy was conducted to investigate the structural properties of GUPS-AuNP. The spectra revealed a broad and strong O-H stretching vibration in the 3400–3300 cm^−1^ range and a weak C-H stretching vibration in the 3000–2800 cm^−1^ range, both characteristic of polysaccharide absorption peaks [13,14]. The absorption peak at 1077 cm^−1^ in GUPS was attributed to the glycosidic bond stretching vibration of the C-O-C bond [15]. Notably, no distinct absorption peak was observed for GUPS-AuNP in this region, which is hypothesized to result from the weakening of the C-O-C bond absorption due to its involvement in the redox reaction during GUPS-AuNP synthesis (Figure 1e). DLS analysis revealed that GUPS-AuNP had a particle size of 120.77 ± 3.13 nm, a zeta potential of −11.9 ± 2.1 mV, and a polydispersity index (PDI) of 0.248 ± 0.015. These results confirmed that GUPS-AuNP is a negatively charged, homogeneous, and well-stabilized nanomaterial (Figure 1f,g).

### 3.2. Maturity and Functionality of GUPS-AuNP-Enhanced DCs

**Figure 2:**
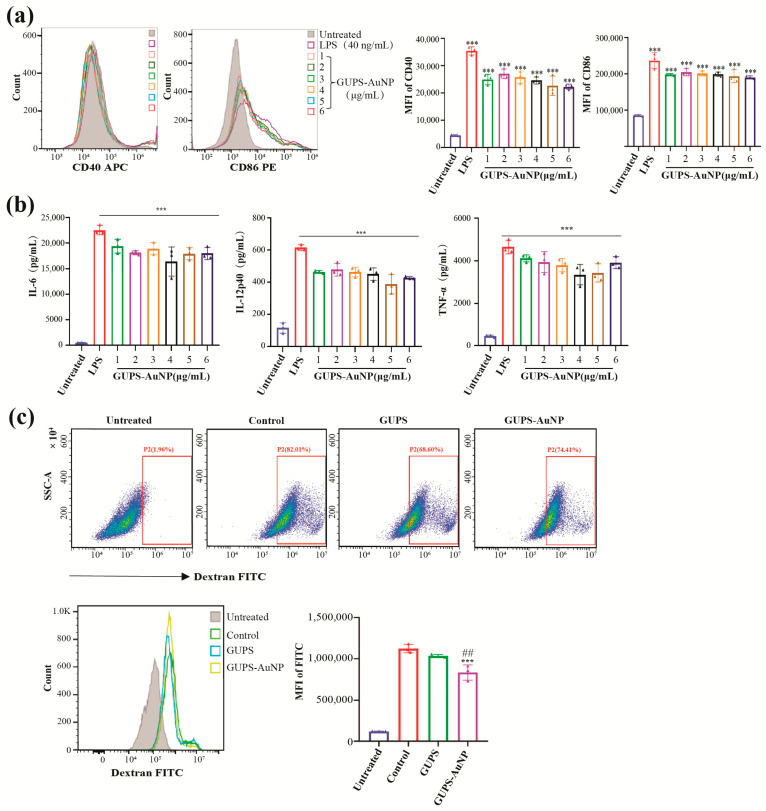
GUPS-AuNP induces maturation and promotes DC function: (**a**) FCM detects the expression of DC surface molecules CD40 and CD86; (**b**) ELISA detects the secretion of IL-6, IL-12p40, and TNF-α (*** *p* ˂ 0.001 compared to untreated); (**c**) DC antigen phagocytosis ability assay, DCs were treated with GUPS-AuNP or GUPS for 12 h, respectively, followed by addition of FTIC-Dextran for 1 h. Flow cytometry was performed to detect the fluorescence intensity of FTIC-Dextran: the proportion of FITC-Dextran^+^ DCs after incubation of GUPS-AuNP-III-treated DCs with FITC-Dextran (top), the ratio of FITC-Dextran relative fluorescence intensity (bottom) (*** *p* ˂ 0.001 compared to untreated; ## *p* < 0.01 compared to GUPS).

To systematically assess the impact of GUPS-AuNP on dendritic cell (DC) maturation and function, DCs were exposed to varying concentrations of GUPS-AuNP (1, 2, 4, 8, 10, and 12 μg/mL), with lipopolysaccharide (LPS) serving as the positive control. After a 12 h treatment, the expression levels of surface molecules and cytokines in DCs were evaluated. The findings revealed that different concentrations of GUPS-AuNP significantly enhanced the expression of DC surface markers CD40 and CD86 (Figure 2a). Additionally, GUPS-AuNP facilitated increased secretion of IL-12p40, TNF-α, and IL-6, thereby promoting DC maturation (Figure 2b).

Mature DCs experience a diminished ability to phagocytize antigens. To confirm DC maturation, we assessed their phagocytic activity. The research results showed that compared with the control group, both GUPS and GUPS-AuNP reduced the proportion of FITC-Dextran^+^ DCs. Interestingly, GUPS-AuNP induced a more pronounced decrease in DC phagocytosis than GUPS alone, implying that GUPS-AuNP may further enhance DC maturation and functional capacity (Figure 2c).

### 3.3. Proliferation of DC-Enhanced T Cells Treated with GUPS-AuNP

**Figure 3:**
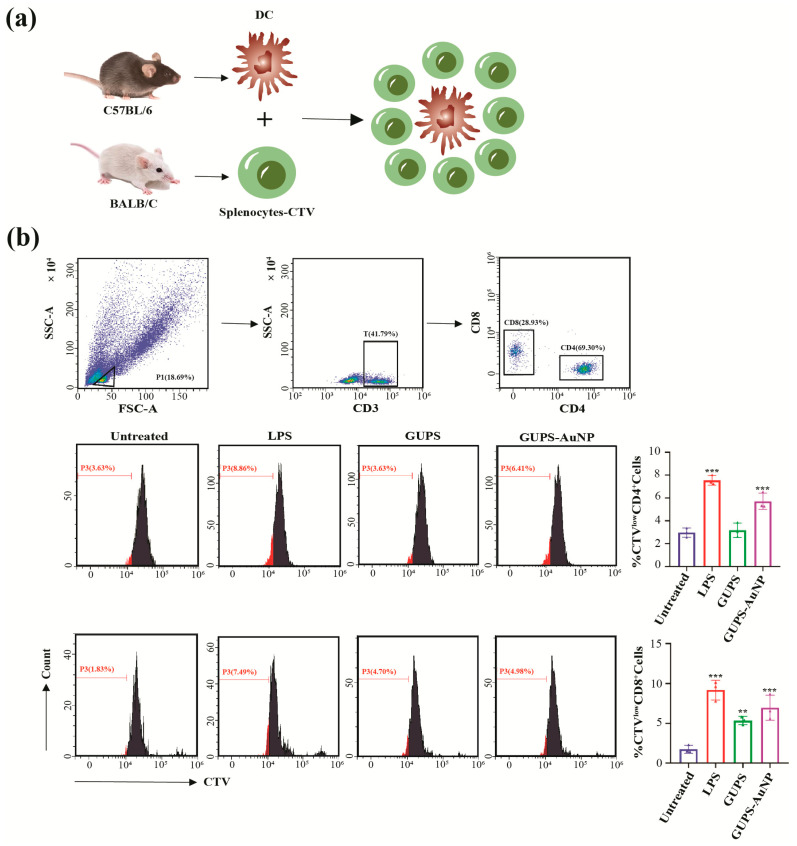
GUPS-AuNP enhanced the function of DCs in promoting T cell proliferation: (**a**) schematic of mixed lymphoid response (MLR); (**b**) effect of GUPS-AuNP-treated DCs on CD4^+^ T and CD8^+^ T proliferation, (** *p* < 0.01, *** *p* ˂ 0.001).

The role of DCs in T cell activation was evaluated using a mixed lymphocyte reaction (MLR) assay (Figure 3a). The results revealed that both GUPS and GUPS-AuNP significantly enhanced the ability of DCs to stimulate the proliferation of CD4^+^ and CD8^+^ T cells. DCs derived from C57BL/6 mice were treated with GUPS-AuNP or GUPS (at a sugar concentration of 2 μg/mL) for 12 h, followed by co-culture with CTV-labeled splenocytes from BALB/c mice at a 1:5 ratio for 72 h. The proliferation of CD4^+^ and CD8^+^ T cells was subsequently assessed. The findings showed that DCs treated with either GUPS or GUPS-AuNP promoted the proliferation of allogeneic CD4^+^ and CD8^+^ T cells to a greater extent compared to the control group. Notably, GUPS-AuNP demonstrated a more pronounced enhancement effect than GUPS (Figure 3b).

### 3.4. GUPS-AuNP Enhances DC Capture of FITC-OVA and Prolongs GUPS-AuNP/FITC-OVA Co-Localization with Lysosomes

DCs are widely recognized as the most potent antigen-presenting cells in the body. To explore the interaction between DCs and antigens, we employed FITC-OVA as a model antigen to evaluate the impact of GUPS-AuNP on the antigen capture ability of DCs. Initially, we investigated the protein-binding properties of GUPS-AuNP. To prepare the GUPS-AuNP/FITC-OVA complex, GUPS-AuNP and FITC-OVA were incubated under dark conditions for 6, 12, and 24 h. The results from UV spectrophotometry and BCA protein quantification revealed that GUPS-AuNP demonstrated optimal binding with FITC-OVA at 6 h, achieving a binding efficiency of 17.5%.

**Figure 4:**
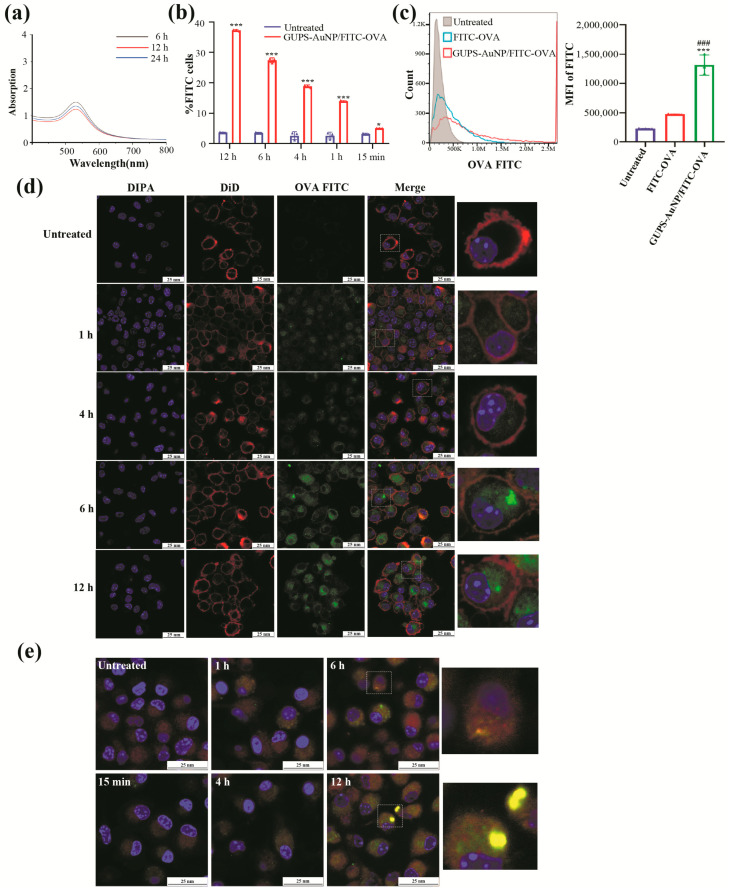
GUPS-AuNP enhances DC capture of antigen and prolongs antigen retention in cells: (**a**) UV scan images of GUPS-AuNP bound to FITC-OVA at different time points; (**b**) ability of GUPS-AuNP/FITC-OVA to enter DCs at different time points; (**c**) FCM assay for 12 h of GUPS-AuNP/FITC-OVA vs. FITC-OVA into DCs (* *p* ˂ 0.05 and *** *p* ˂ 0.001 compared to untreated; ### *p* ˂ 0.001 compared to FITC-OVA); (**d**) CLSM antigen delivery assay of GUPS-AuNP at different time points, The rightmost panel displays the enlarged region marked by the white dashed line; (**e**) CLSM assay for localization of GUPS-AuNP delivered antigen to lysosomes at different times, The rightmost panel displays the enlarged region marked by the white dashed line.

By incubating GUPS-AuNP/FITC-OVA with DCs for varying durations, we observed a time-dependent increase in the internalization of GUPS-AuNP/FITC-OVA by DCs, with the fluorescence intensity reaching its peak at 12 h (Figure 4b). A further comparison of the internalization efficiency between GUPS-AuNP/FITC-OVA and FITC-OVA following 12 h of incubation revealed that the GUPS-AuNP/FITC-OVA group exhibited significantly higher relative fluorescence than the FITC-OVA group (Figure 4c).

To better assess the ability of GUPS-AuNP to enhance antigen capture by DCs, we performed confocal laser scanning microscopy (CLSM) at various incubation intervals. After 1 h of incubation, green fluorescence representing antigen entry into the cells became visible, as illustrated in the figure. The fluorescence intensity increased proportionally with the amount of antigen taken up by the DCs, a result further validated through flow cytometry analysis. These findings clearly demonstrate that GUPS-AuNP substantially enhances the efficiency of antigen capture by DCs.

In our previous studies, we demonstrated that GUPS-AuNP significantly enhances the antigen-capturing ability of DCs. To further explore the intracellular distribution of captured antigens, we varied the incubation times, stained lysosomes using Lyso-Tracker Red, and analyzed the co-localization of GUPS-AuNP/FITC-OVA with lysosomes. CLSM revealed that GUPS-AuNP/FITC-OVA co-localized with lysosomes only after 6 h. This observation suggests that GUPS-AuNP/FITC-OVA does not rapidly enter lysosomes for degradation but instead extends its retention within the cells, thereby promoting antigen cross-presentation (Figure 4e).

### 3.5. GUPS-AuNP-DC + HPV Inhibits Tumor Growth

**Figure 5:**
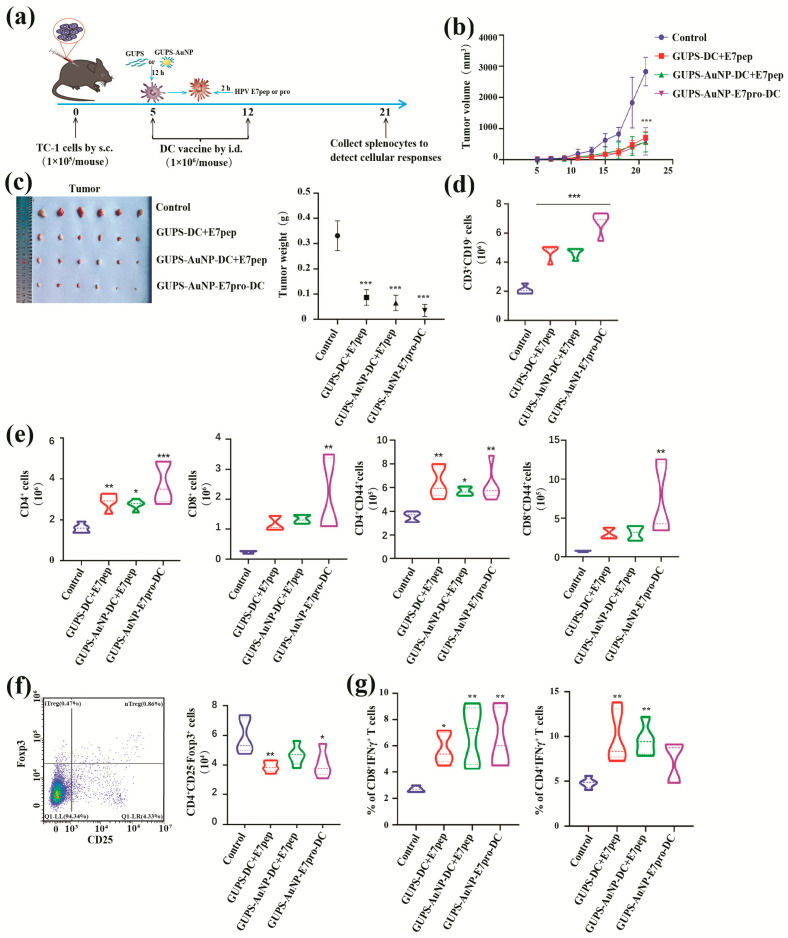
In vivo antitumor effects: (**a**) TC-1 tumor model establishment and treatment strategy; (**b**) tumor volume and area under the curve (AUC); (**c**) tumor photography and weighing results on day 21; (**d**) number of T cells; (**e**) number of CD4^+^ T and CD8^+^ T cells and T cell activation; (**f**) number of iTregs cells; (**g**) HPV antigen-specific T cell detection of response (* indicates * *p* < 0.05, ** *p* < 0.01, *** *p* ˂ 0.001 compared to untreated).

To assess the antitumor efficacy of the HPV-DC vaccine using GUPS-AuNP as a carrier, we established a TC-1 mouse tumor model (Figure 5a). Compared to the control group, the vaccine-treated group exhibited a marked suppression of tumor growth (Figure 5b) and a significant reduction in tumor weight (Figure 5c). Among the vaccine-treated groups, the GUPS-AuNP + E7pro-DC group demonstrated the most robust tumor-suppressive effect. All vaccine-treated groups, in contrast to the control group, showed an increase in the number of CD3^+^ T cells, CD4^+^ T cells, and CD8^+^ T cells, as well as their activation markers (CD8^+^CD44^+^, CD4^+^CD44^+^). Notably, the GUPS-AuNP + E7pro-DC group displayed the highest levels of T cell activation (Figure 5d,e). Moreover, the proportion of immunosuppressive cells (iTregs, CD4^+^CD25-Foxp3^+^) was significantly diminished in the vaccine-treated groups (Figure 5f). These findings suggest that the vaccine elicits a strong antitumor immune response in mice, primarily by activating T cells.

To assess the specific immune response triggered by the vaccine, we stimulated spleen cells from each group in vitro with the HPV E7 peptide for 10 h and analyzed the proportions of CD4^+^ IFN-γ^+^ and CD8^+^IFN-γ^+^ T cells. The results revealed that all vaccine groups induced antigen-specific T cell immune responses in the spleens of the mice, with the GUPS-AuNP + E7pro-DC group exhibiting the most pronounced effect (Figure 5g). GUPS-AuNP proved to be an effective carrier for delivering HPV protein antigens, significantly enhancing the vaccine-induced antigen-specific immune response. Additionally, the protein antigen demonstrated markedly superior efficacy compared to the peptide antigen.

## 4. Discussion

With advancements in nanotechnology, nanomaterials have found widespread applications across diverse fields, such as biological carriers, disease diagnosis, and food safety inspection. Among the various nanomaterials, AuNPs stand out due to their relatively straightforward synthesis process, ease of functionalization, and remarkable chemical and physical properties. These attributes enable AuNPs to play pivotal roles in drug delivery, photothermal therapy, and biosensing [16,17]. AuNPs can be synthesized using multiple approaches, including chemical, physical, and biological methods, with citrate being the most commonly utilized reducing agent for optimizing the synthesis of AuNPs with varying particle sizes [18]. While chemical synthesis methods for AuNPs are well-established, they may present potential health risks, underscoring the need to explore safer and more sustainable alternatives. Recently, there has been increasing interest in the green synthesis of AuNPs using biological systems such as plants, bacteria, yeast, and fungi [19,20]. Certain plants, including their roots, stems, and leaves, exhibit notable reducing properties, making them effective reducing and stabilizing agents for AuNP synthesis [21]. Polysaccharides, as vital biological macromolecules, are abundantly present in animals, plants, and microorganisms. They possess significant immunomodulatory activity, supporting humoral, cellular, and mucosal immune responses, and hold considerable promise for treating tumors, infections, and autoimmune diseases [22]. Polysaccharide molecules contain numerous reducing hydroxyl groups, which are more accessible in low-molecular-weight polysaccharides. This property makes natural polysaccharides efficient reducing agents for the synthesis of AuNPs [23]. Noruzi et al. successfully synthesized AuNPs using rose petal extract, with qualitative analysis confirming a high sugar content capable of effectively reducing Au^3+^, highlighting the potential of polysaccharides as promising reducing agents in the green synthesis of gold nanoparticles [24]. In the current study, GUPS extracted in our laboratory were utilized as reducing agents to green-synthesize GUPS-AuNPs. The physicochemical properties of GUPS-AuNPs were subsequently characterized, displaying excellent dispersibility and stability. These findings enrich the biosynthesis of AuNPs and provide a theoretical foundation for advancing the green synthesis of AuNPs.

Polysaccharide-based gold nanomaterials present numerous advantages, such as enhanced immune response activation and prolonged in vivo retention, making them extensively applied in the biological sciences [25]. In this study, the synthesized GUPS-AuNPs markedly increased the expression of CD40 and CD86 on the surface of dendritic cells (DCs) and stimulated the secretion of cytokines IL-12p40, TNF-α, and IL-6. These results indicate that GUPS-AuNPs effectively promote DC maturation while simultaneously reducing their antigen phagocytosis capacity. Compared to GUPS alone, DCs treated with GUPS-AuNPs exhibited a superior ability to induce T cell proliferation and activation, signifying enhanced immunomodulatory activity. This observation aligns with the findings by Zhang et al. [26], who synthesized Ganoderma lucidum polysaccharide gold nanoparticles (GLP-AuNPs), and Peng et al. [27], who developed Astragalus polysaccharide gold nanoparticles (APS-AuNPs).

DCs, as professional antigen-presenting cells (APCs), play a pivotal role in initiating antigen-specific T cell responses. DC-based cancer vaccines have attracted considerable interest from researchers and hold immense potential in advancing tumor immunotherapy [28]. Nevertheless, several challenges hinder their efficacy, such as insufficient antigen presentation, limited migratory capacity, and inadequate cytokine release. Thus, fostering DC maturation, enhancing T cell proliferation, and improving antigen presentation are crucial strategies for boosting the effectiveness of DC vaccines [29]. Optimizing antigen presentation systems can substantially amplify the performance of these vaccines. Compared to conventional pharmaceuticals, nanomaterials offer superior targeting capabilities and enhanced delivery efficiency [30]. Among these, gold nanoparticles (AuNPs) have emerged as promising tools for antigen delivery due to their unique properties. Specifically, boronic acid-engineered gold nanoparticles have demonstrated an ability to efficiently deliver multiple proteins into cells while preserving their biological activity [31]. In this study, the synthesized GUPS-AuNPs significantly enhanced the uptake of ovalbumin (OVA) protein by DCs and prolonged its retention within cells, thereby facilitating robust antigen cross-presentation.

Tumor antigens exist in various forms, including peptides, nucleic acids, proteins, and tumor lysates, each of which directly influences the specificity and intensity of immune responses induced by vaccines. Nucleic acid antigens (RNA or DNA) enter host cells via mechanisms such as membrane permeability, endocytosis, or membrane fusion. Once inside, they are expressed using the host’s cellular machinery, primarily activating CD8 T cell responses as endogenous antigens. However, their use is limited due to issues such as poor stability, susceptibility to degradation, and low immunogenicity [32,33]. Peptide antigens, consisting of 10 to 20 amino acids, are recognized by MHC molecules and presented on the cell surface, where they interact with T cells in the immune system to initiate an immune response. These antigens primarily activate CD4 T cell responses as exogenous antigens [34]. Peptide antigens come with advantages like low cost, easy access, and single specificity, but they also face challenges such as MHC restriction and limited stability [35]. In contrast, whole protein antigens offer unique benefits, including modifiability, broad-spectrum antigenicity, and independence from MHC restriction, making them widely applied in vaccine development [36]. Nonetheless, their broader application is hindered by complex preparation and purification processes, stringent storage conditions, and vulnerability to contamination. Despite these challenges, this study successfully prepared GUPS-AuNPs as carriers for HPV DC vaccines and evaluated their therapeutic effects on tumors. The DC vaccines, employing GUPS-AuNPs to deliver HPV peptide and protein antigens, demonstrated significant tumor growth inhibition while activating T cells and generating robust antitumor immune responses. Among the combinations tested, the GUPS-AuNP + E7pro-DC formulation exhibited the most pronounced inhibitory effect on tumor growth. Specific immune responses were elicited by all vaccines, with antigen-specific T cell activation observed in the spleens of mice. Once again, the GUPS-AuNP + E7pro-DC group showed the strongest impact. In conclusion, GUPS-AuNPs effectively function as carriers for HPV peptide and protein antigens, enhancing the antigen-specific immune responses induced by the vaccines. Notably, protein antigens demonstrated superior efficacy compared to peptide antigens in this study.

## 5. Conclusions

In summary, this study utilized GUPS as a reducing agent to synthesize GUPS-AuNPs, which significantly promoted the maturation and functionality of DCs. This method enhanced DCs’ ability to capture OVA protein and extended the intracellular retention time of the protein, thereby facilitating efficient cross-presentation of the antigen. Moreover, the HPV DC vaccine formulated with GUPS-AuNPs as a carrier triggered a robust antitumor immune response in vivo. Although GUPS-AuNPs effectively enhance immune responses, several limitations remain. Their bioavailability and in vivo stability require optimization to improve clinical applicability. Nanoparticle size and surface modifications also influence immune cell interactions and antigen delivery efficiency. Future studies should focus on three key areas: refining GUPS-AuNP synthesis methods to optimize the particle size and surface properties for enhanced stability and biodistribution; investigating synergistic effects with other immunopotentiators or therapies to boost immune activity; and assessing long-term efficacy and safety in preclinical models to support clinical translation. These optimizations could establish GUPS-AuNPs as a safer, more efficient platform for tumor immunotherapy and vaccine development.

## Data Availability

The original contributions presented in the study are included in the article/Supplementary Materials, further inquiries can be directed to the corresponding author.

## Supplementary Materials

The following supporting information can be downloaded at: https://www.mdpi.com/article/10.3390/pharmaceutics17091213/s1, Figure S1: preparation, characterization, and effect of GUPS-AuNPs on DCs: (**a**) green preparation process of GUPS-AuNPs; (**b**) GUPS-AuNPs prepared with different ratios of GUPS and corresponding UV scan profiles; (**c**) SEM images of GUPS-AuNPs; (**d**) FCM detection of CD40 and CD86 expression on the surface of DCs; (**e**) ELISA detection of IL-6, IL-12p40, and TNF-α secretion (* indicates ** *p* < 0.01, *** *p* ˂ 0.001 compared with untreated; # indicates ## *p* < 0.01, ### *p* ˂ 0.001 compared with GUPS).

## Abbreviations

The following abbreviations are used in this manuscript:

HPV	Human papillomavirus
GUPS	Glycyrrhiza polysaccharide
DC	Dendritic cell
GUPS-AuNP	Glycyrrhiza polysaccharide gold nanoparticles
AuNP	Gold nanoparticles
FTIR	Fourier transform infrared
DLS	Dynamic light scattering
PDI	Polydispersity index
MLR	Mixed lymphocyte reaction
CLSM	Confocal laser scanning microscopy
GLP-Au	Ganoderma lucidum polysaccharide gold nanoparticles
APCs	Antigen-presenting cells
OVA	Ovalbumin
