# Supplementary material for: Glycyrrhiza uralensis Polysaccharide Gold Nanoparticles as Antigen Carriers and Potential Adjuvant to DC Vaccines
Source: Pharmaceutics. 2025 Sep 17;17(9):1213. doi: 10.3390/pharmaceutics17091213 (PMC12473970; doi:10.3390/pharmaceutics17091213)
Supplement: Supplementary file 1 [file pharmaceutics-17-01213-s001.zip › pharmaceutics-3711421-supplementary.pdf]

Supplementary Materials: The following supporting information can be downloaded, Figure S1: Synthesis, characterisation and effect of GUPS-AuNPs on DC.

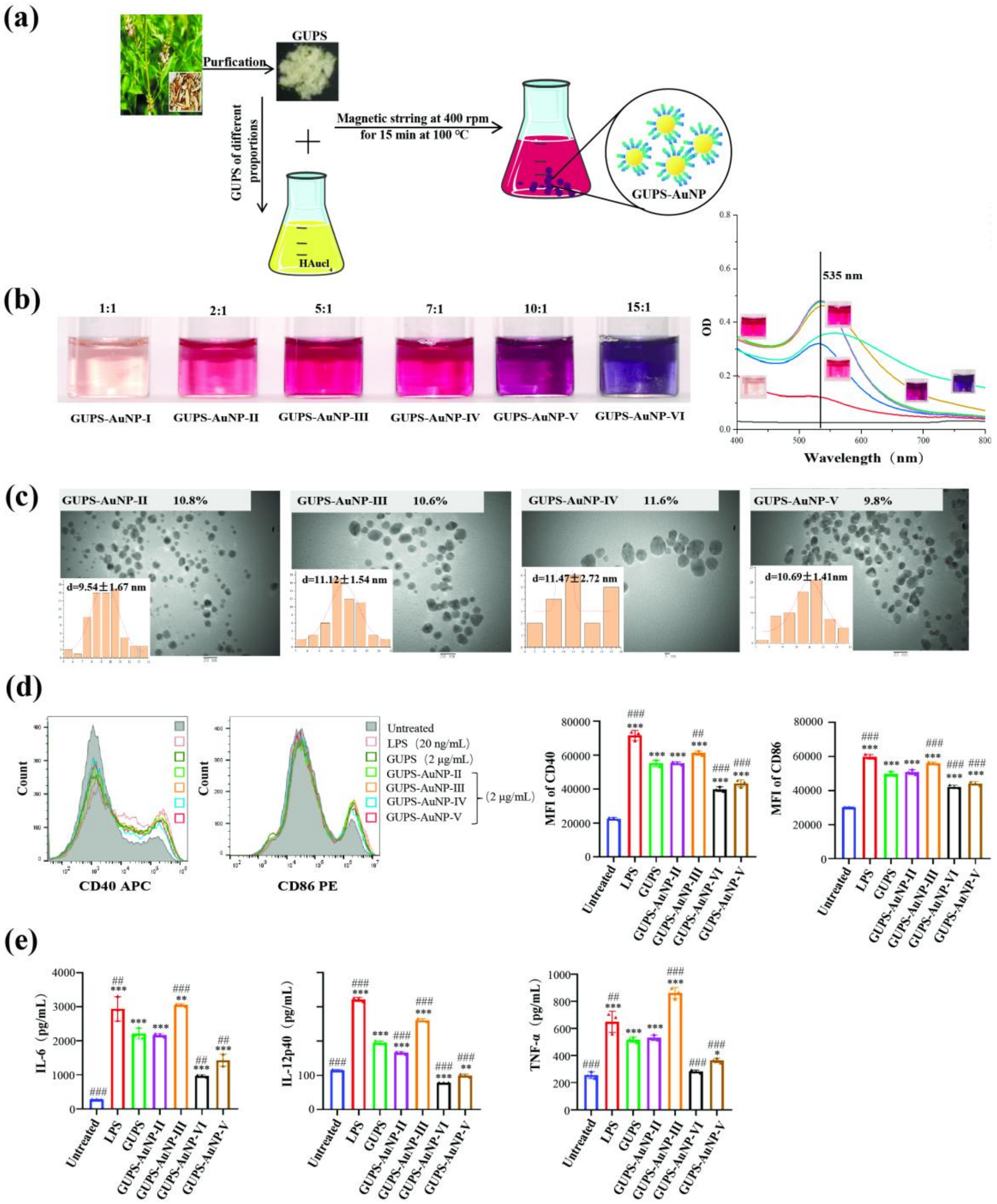

Figure S1. Preparation, characterisation and effect of GUPS-AuNPs on DC: (**a**) Green preparation process of GUPS-AuNPs; (**b**) GUPS-AuNPs prepared with different ratios of GUPS and corresponding UV scan profiles; (**c**) SEM images of GUPS-AuNPs; (**d**) FCM detection of CD40 and CD86 expression on the surface of DCs; (**e**) ELISA detection of IL-6, IL-12p40 and TNF-α secretion (* indicates **P<0.01, ***P<0.001 compared with untreated; # indicates ##P<0.01, ##P<0.001 compared with GUPS).
